# Frequencies of HER-2/neu expression and gene amplification in patients with oesophageal squamous cell carcinoma

**DOI:** 10.1038/sj.bjc.6602499

**Published:** 2005-03-22

**Authors:** K Mimura, K Kono, M Hanawa, F Mitsui, H Sugai, N Miyagawa, A Ooi, H Fujii

**Affiliations:** 1 First Department of Surgery, University of Yamanashi, 1110 Tamaho, Yamanashi 409-3898, Japan; 2 First Department of Pathology, University of Yamanashi, Yamanashi 409-3898, Japan

**Keywords:** HER-2/neu, FISH, immunohistochemistry, HLA, immunotherapy

## Abstract

The utilisation of antitumour T cells induced by cancer vaccination with HER-2 peptides or antibodies (Herceptin) against HER-2, as immunotherapy for oesophageal cancer, is a novel and attractive approach. It is important to clarify the frequencies of HER-2 expression and gene amplification in patients with oesophageal squamous cell carcinoma (SCC) and to evaluate the relationship between HER-2 status and HLA haplotype, since the candidates for HER-2 peptide-based vaccination are restricted to a certain HLA haplotype. We determined the frequency of HER-2 expression using the HercepTest™ for immunohistochemistry and HER-2 gene amplification by fluorescence *in situ* hybridisation (FISH) assay in oesophageal SCC (*n*=66). HER-2-positive tumours (1+/2+/3+) analysed by a HercepTest were observed in 30.3% of all the patients and HER-2 gene amplification evaluated by FISH was observed in 11.0% of all the patients, in which all HercepTest (3+) tumours were found to have gene amplification and three of six moderately positive (2+) tumours showed gene amplification. Furthermore, HER-2-positive cells were present more diffusely and were larger within each tumour in the patients who were HercepTest 3+ than those who were HercepTest 1+. Moreover, the survival rate in HER-2-positive group was significantly worse than that in HER-2-negative group. Also, the survival rate in the patients with HER-2 gene amplification was significantly worse than that without HER-2 gene amplification. In addition, oesophageal SCC patients with both HLA-A24-positive and HER-2-positive tumours (1+/2+/3+) accounted for 26% of these cases, and both HLA-A2- and HER-2-positive tumours accounted for 18% of them.

While most patients with oesophageal cancer in Western countries have adenocarcinoma, most of those in Japan have squamous cell carcinoma (SCC). Despite various treatments such as surgical resection with extensive lymphadenectomy (Maillet *et al*, 1982; Altorki and Skinner, 1990; Kato *et al*, 1991; Akiyama *et al*, 1994) and the surgery combined with chemotherapy (Hayashi *et al*, 2001) and/or radiotherapy (Le Prise *et al*, 1995; Adham *et al*, 2000; Ishikura *et al*, 2003), the prognosis for advanced patients with oesophageal SCC remains poor. The utilisation of antitumour T cells or antibodies against tumour antigens, as immunoadjuvant therapy for oesophageal SCC, is therefore an attractive approach.

The HER-2/neu (designated as HER-2) proto-oncogene located on chromosome 17(17q12–q21.32) (Popescu *et al*, 1989) encodes a 185-kDa transmembrane glycoprotein with tyrosine-specific kinase activity (Coussens *et al*, 1985). The HER-2 proto-oncogene is amplified and overexpressed in approximately 30% of human ovarian and breast tumours (Slamon *et al*, 1989), and in 8.2% of gastric cancers (Takehana *et al*, 2002). The humanised monoclonal antibody (mAb) Herceptin, which specifically targets HER-2, exhibits potent growth inhibitory activity against HER-2-over expressing tumours (Sliwkowski *et al*, 1999). Herceptin has boosted the interest of clinicians in immunotherapy based on this molecule as it represents the first mAb approved for therapeutic use with proved survival benefit in patients with HER-2-positive breast cancer with metastasis (Baselga *et al*, 1996; Slamon *et al*, 2001). Moreover, abundant examples from experimental models and clinical trials suggest that HER-2 can be immunogenic and generate antibodies, CTL- and helper T-cell-specific responses in individuals with HER-2-overexpressing tumours (Fisk *et al*, 1995; Kono *et al*, 1998). Based on the above reports, anti-HER-2 immune targeting could be utilised as an attractive approach to treat oesophageal cancer. Thus, it is important to clarify the frequency of HER-2 expression and gene amplification relating to the HLA haplotype in order to determine possible candidates for HER-2-based immunotherapy, since the candidates for HER-2 peptide-based vaccination are restricted to a certain HLA haplotype.

With respect to oesophageal SCC, the frequencies of HER-2 overexpression analysed by immunohistochemistry (IHC) ranged from 0 to 55.9% (Mori *et al*, 1987; Chang *et al*, 1992; Suo *et al*, 1992; Shiga *et al*, 1993; Suwanagool *et al*, 1993; Suo *et al*, 1995; Hardwick *et al*, 1997; Lam *et al*, 1998; Akamatsu *et al*, 2003). Furthermore, reports describing HER-2 gene amplification ranged from 0 to 25%, and these studies were performed by Northern blot, slot blot or RT–PCR analysis (Shiga *et al*, 1993; Ikeda *et al*, 1996; Tanaka *et al*, 1997; Friess *et al*, 1999). The discrepancy in the HER-2 frequencies among the reports may be related to the methodology, including the different mAbs used in IHC or inaccurate analysis for gene amplification. There has been no previous report describing HER-2 gene amplification in oesophageal SCC analysed by fluorescence *in situ* hybridisation (FISH) analysis.

In the present study, we determined the exact frequency of HER-2 abnormalities using the HercepTest™ for IHC and the PathVysion test for FISH in oesophageal SCC, and analysed patient's data for the survival rate. Both the HercepTest and the PathVysion FISH assay are approved by the US Food and Drug Administration (FDA) for determining the eligibility for Herceptin treatment in breast carcinoma. Furthermore, we have evaluated a possible candidate for anti-HER-2 immune targeting therapy for oesophageal SCC.

## MATERIALS AND METHODS

### Patients and samples

In all, 66 consecutive patients with primary oesophageal SCC who were histologically diagnosed and treated in the First Department of Surgery, University of Yamanashi Hospital, between 1998 and 1999, were enrolled in the present study and all the patients were followed up for 5 years. None of the patients had received any treatment before surgery (preoperative radiotherapy or chemotherapy) and all patients had undergone oesophagectomy with two-field (*n*=39) or three-field (*n*=27) lymph node dissection. The patients were classified using the tumour node metastasis (TNM) classification. The characteristics of the patients are shown in Table 1. The study was approved by the ethical committee of University of Yamanashi and written informed consent was obtained from all individuals.

Formalin-fixed, paraffin-embedded tissue blocks were used for IHC and FISH analysis.

### HLA class I typing

Heparinised peripheral blood was obtained from patients prior to the operation. Peripheral blood lymphocytes (PBLs) were purified by centrifugation on a Ficoll gradient (Pharmacia, Uppsala, Sweden). For class I typing, PBLs were subjected to a complement-dependent microcytotoxicity assay using antisera to HLA-A loci. Peripheral blood lymphocytes were typed for A loci 1, 2, 3, 9, 10, 11, 19, 23, 24, 25, 26, 28, 29, 30, 31, 32, 33, 34, 36, 43, 66, 68, 69 and 74.

### IHC analysis

**Figure 1:**
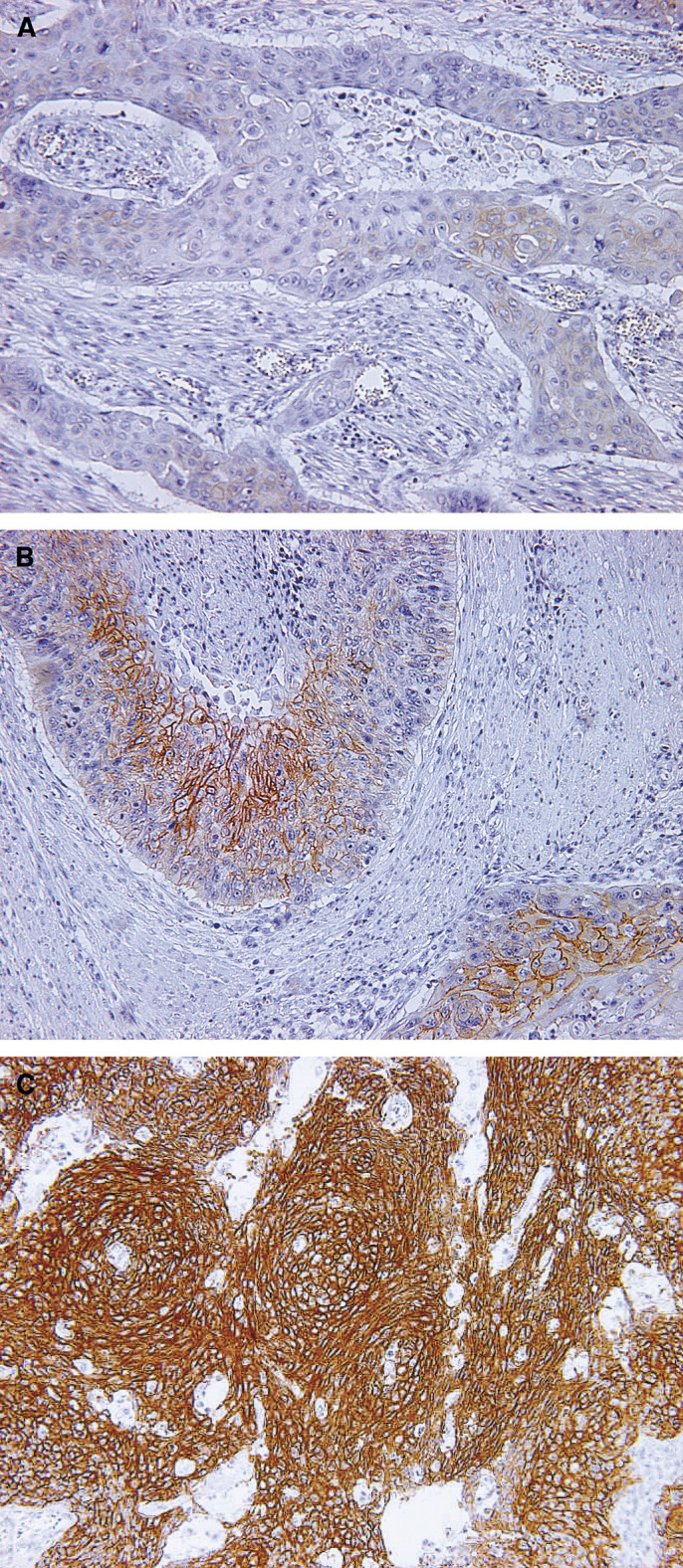
Representative immunostaining of HER-2-positive cells. (**A**) 1+ staining cases, (**B**) 2+ staining cases and (**C**) 3+ staining cases. Original magnification × 200.

Immunohistochemical staining was performed using the HercepTest™ (DaKoCytomation, Denmark) according to the manufacturer's recommendations. Archival, formalin-fixed, paraffin-embedded material was used to obtain 4-*μ*m-thick sections from the main tumour and the regional lymph nodes. Briefly, deparaffinised and rehydrated tissue sections were incubated with the Epitope Retrieval Solution in a heat water bath for 40 min at 95–99°C. Then, the sections were cooled at room temperature for 20 min and washed with TRIS buffer for 5 min. Next, endogenous peroxidase was blocked with 3% hydrogen peroxide for 5 min. The primary antibody was a rabbit polyclonal antibody to human HER-2, which recognises an intracytoplasmic part of HER-2, and the primary negative control antibody was an immunoglobulin fraction of normal rabbit serum at an equivalent protein concentration as the antibody to HER-2. The sections were washed with a TRIS buffer for 5 min and incubated with the primary antibody or the primary negative control antibody at room temperature for 30 min. After rewashing with a TRIS buffer for 5 min × 2 times, the primary antibody was detected using the Visualisation Reagents, which were a dextran polymer conjugated with horseradish peroxidase and affinity-isolated goat anti-rabbit immunoglobulins, for 30 min of incubation at room temperature. Subsequently, following rewashing with TRIS buffer for 5 min × 2 times, diaminobenzidine was added as a visualisation reagent for 10 min and the section was counterstained with haematoxylin. Control slides provided with the HercepTest™ kit, which contained three human breast cancer cell lines with staining intensity scores of 0, 1+ and 3+, were used in the present study. IHC analysis was performed by two observers (KM and KK) according to the staining intensity scores provided by the HercepTest™ kit. Each section was classified into four categories (0, 1+, 2+, 3+), in which tumour cells with complete absence of staining were scored as 0, those with incomplete membranous staining were classified as 1+, those with moderate, complete membranous staining were classified as 2+ and those with strong, complete membranous staining were classified as 3+ (Figure 1).

**Figure 2:**
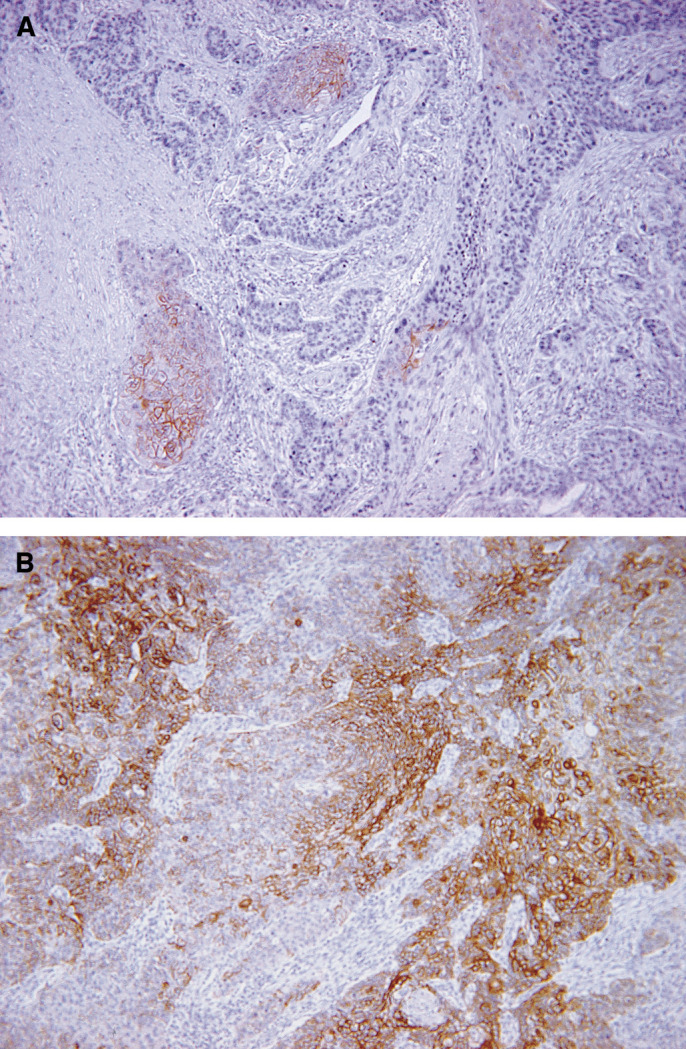
Heterogeneity in the pattern of HER-2 immunostaining. The staining pattern was categorised as spot type and diffuse type. (**A**) Spot type and (**B**) diffuse type. Original magnification × 100.

Furthermore, we evaluated the immunostaining pattern into two patterns, spot type or diffuse type (Figure 2), in which the diffuse type indicated tumour cells with membranous staining spread throughout the tumour tissue continuously. On the other hand, the spot type indicated tumour cells with membranous staining on one part or several parts separately in the section.

### FISH analysis

FISH analysis was performed using the PathVysion® HER-2 DNA Probe Kit (VYSIS, Downers Grove, IL, USA). The HER-2/neu-SpectrumOrange probe is specific for the HER-2 gene locus (17q11.2–q12). The CEP 17 (chromosome enumeration probe)/SpectrumGreen probe is specific for the alpha-satellite DNA sequence (centromere region of chromosome 17). To determine the copy number for chromosome 17, we used CEP 17 as the control. FISH procedures were conducted according to the manufacturer's guidelines, except the removal of the protein from the section where we used our own protocol as described previously (Takehana *et al*, 2002). Briefly, sections were deparaffinised, dehydrated and incubated in 20% sodium bisulphate/2 × standard saline citrate at 43°C for 20 min. Sections were washed with SCC and treated with proteinase K (Boehringer-Mannheim, Mannheim, Germany) at 37°C for 25 min. Subsequently, denaturation, hybridisation and posthybridisation washing were performed according to the manufacturer's guidelines, and after hybridisation and posthybridisation washing, the sections were counterstained with DAPI (4′,6-diamidine-2′-phenylindole dihydrochloride). FISH analysis was performed using a fluorescence microscope (Olympus, Tokyo, Japan) equipped with Triple Bandpass Filter sets (Vysis). Signals were counted for at least 40 cancer nuclei per tumour. In accordance with earlier studies with FISH, a cell was considered to show amplification when a definite cluster or more than 10 orange signals of HER-2 was observed (Takehana *et al*, 2002). A positive control, which is breast tumour with previously identified HER-2 amplification and overexpression, was used as a positive control for HER-2 FISH.

### Statistical analysis

The *χ*^2^ test was applied to examine the differences in frequencies of the HLA-A haplotype and HER-2 expression in oesophageal SCC, the differences in HercepTest score and the rate of HER-2-positive cells in each tumour, and the differences in the lymph node metastasis of HER-2-positive patients and HER-2-negative patients. Actuarial overall survival rates were analysed by the Kaplan–Meier method and survival was measured in months from operation to death or last review. The log-rank test was applied to compare with the two groups. Univariate and multivariate survival analysis were calculated according to Cox's proportional-hazards model. All statistical analyses were performed using Statview 5.0 for Windows software and statistically significant difference was considered as *P*-values <0.05.

## RESULTS

### Frequencies of HER-2 expression and gene amplification

We studied 66 oesophageal SCC tumours and their regional lymph nodes. In IHC, each section was classified into four categories (negative, 1+, 2+, 3+) according to the staining intensity scores provided by the HercepTest kit. Positive immunostaining (1+/2+/3+) of HER-2 expression was found in 20 (30.3%) of the 66 patients with oesophageal SCC (Table 2). The clinicopathological data and their FISH analysis are summarised in Table 3. Three patients (4.5%) showed strong positive staining (3+) and 6 (9.1%) showed moderate positive staining (2+).

**Figure 3:**
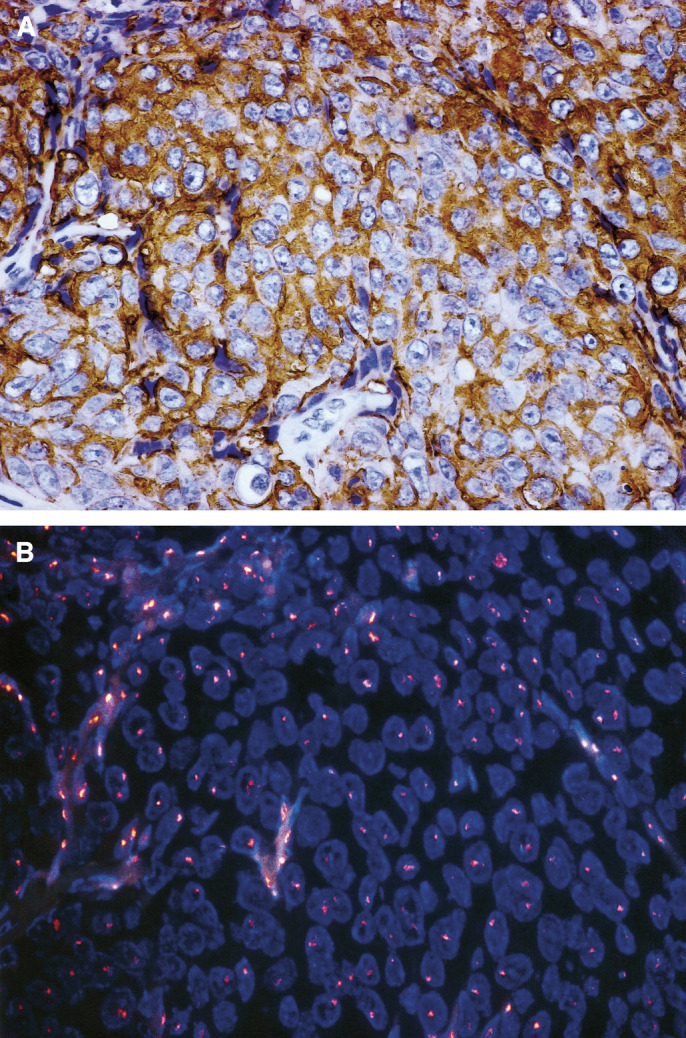
Representative FISH analysis of HER-2 gene amplification (cluster formation). The serial sections in the representative case were analysed by IHC (**A**) (3+ staining cases, × 400) and FISH analysis (× 400 in (**B**)).

With respect to FISH, a cell was considered to show amplification when a definite cluster or more than 10 orange signals of HER-2 was observed in accordance with earlier studies with FISH (Takehana *et al*, 2002). In FISH analysis for IHC-positive oesophageal SCC (*n*=20), HER-2 gene amplification (cluster, Figure 3) was found in seven tumours (Table 3). In the three tumours, cancer nuclei showed more than three HER-2 signals accompanied with the same number of centromere 17 signals. They were judged as polysomy 17 (Table 3).

With respect to the comparison of FISH and IHC analysis, all the strong positive (3+) tumours were found to have gene amplification, as shown in Table 3. Among six moderate positive (2+) tumours, three showed gene amplification (cluster) and one showed polysomy. In 11 weak positive (1+) tumours, one showed gene amplification (cluster) and two showed polysomy.

Taken together, positive immunostaining (1+/2+/3+) for HER-2 expression was found in 30.3%. Moreover, moderate and strong positive patients (2+/3+) had a high frequency of gene amplification, while weak positive (1+) patients showed a low frequency of gene amplification.

### Immunostaining pattern and rate of HER-2-positive cells within each tumour

When the clinical application of anti-HER-2 immune targeting is considered, it is important to clarify the heterogeneity in the pattern of HER-2-positive tumour cells within each tumour. According to the DAKO HercepTest kit, the grading of the HercepTest depends on the intensity of membranous staining, indicating that the grading does not reflect how many cells are HER-2 positive. Thus, in the present study, we evaluated the immunostaining pattern and rate of HER-2-positive cells within the tumour.

We recognised that there was heterogeneity in the pattern of HER-2 immunostaining and categorised the staining pattern into a spot type and diffuse type (Figure 2). Also, HER-2-positive cells were semiquantified by counting the average number in each tumour. As a result, all three strong positive (3+) cases showed the diffuse-type pattern (Table 3) and had HER-2-positive cells in more than 30% of tumours (Table 4), while most of the weak positive (1+) patients showed that HER-2-positive cells were less than 30% (Table 4) (*P*=0.057, *χ*^2^ analysis). Thus, HER-2-positive cells were present more diffusely within each tumour in the HercepTest 3+ patients than those who were HercepTest 1+.

### Correlation of HER-2 expression in primary tumours and metastatic lymph nodes

Of all 66 cohorts, 37 (56.1%) had metastatic lymph nodes diagnosed by histopathological determination. While 15 (75.0%) in the 20 HER-2-positive patients had metastatic lymph nodes, 22 (47.8%) in the 46 HER-2-negative patients had metastatic lymph nodes (Table 3), indicating that there was a high frequency of lymph node metastasis in the HER-2-positive patients in comparison to HER-2-negative patients (*P*<0.05, *χ*^2^ analysis). All the strongly positive (3+) patients in the primary tumours were found to have HER-2-positive tumours (3+) in the metastatic lymph nodes, while there was only one patient with a HER-2-positive tumour in the metastatic lymph node in the weak positive (1+) patients with lymph node metastasis (*n*=8).

### Frequency of the HLA haplotype relating to HER-2 status

When anti-HER-2 immunotherapy such as cancer vaccination is considered for HER-2-positive patients, the candidates are restricted to a certain HLA haplotype (Kono *et al*, 2002a). It is important to clarify the HER-2 status relating to the HLA haplotype in patients with oesophageal SCC. In the patients tested for HLA haplotypes (*n*=50), the distribution of the HLA-A haplotype in patients with oesophageal SCC is shown in Table 5. The most frequent HLA-A haplotypes in oesophageal SCC are HLA-A24 (64%), HLA-A2 (52%) and HLA-A11 (28%). Also, oesophageal cancer patients with both HLA-A24-positive and HER-2-positive tumours accounted for 26% of these cases, and both HLA-A2-positive and HER-2-positive tumours accounted for 18% of them. There was no significant relation between the frequency of the HLA-A haplotype and the HER-2 status (Table 5).

### Analysis of the survival of patients with oesophageal SCC

**Figure 4:**
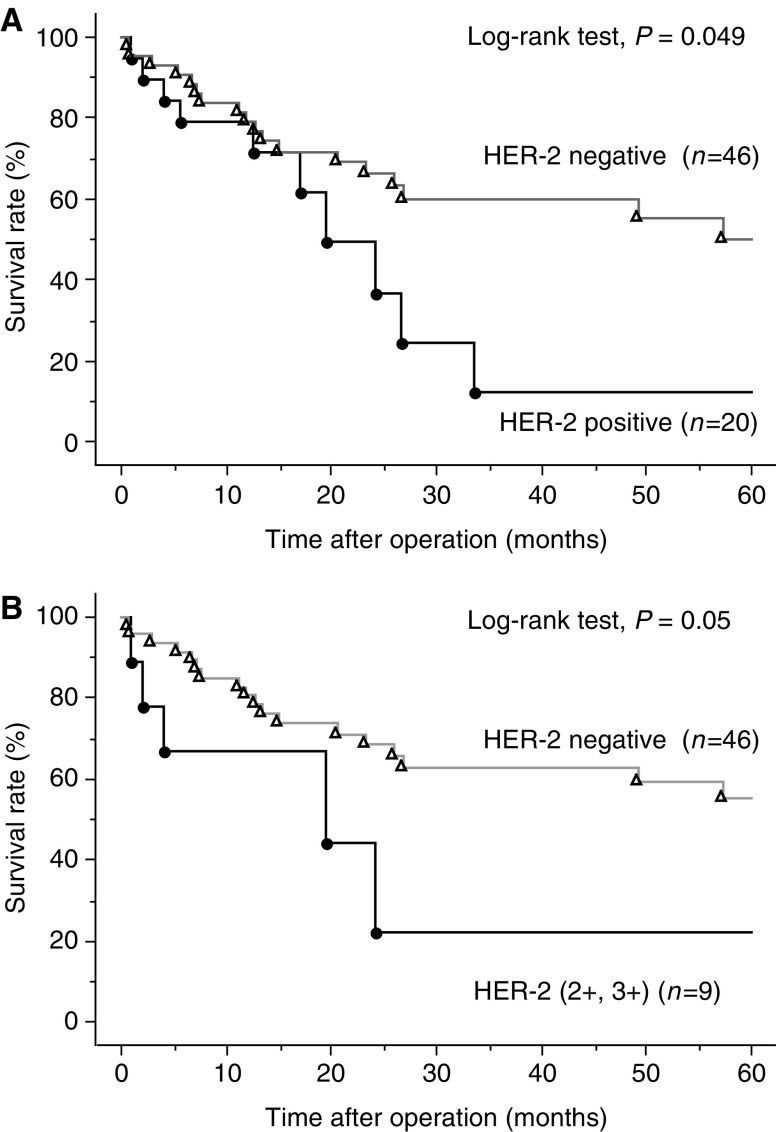
(**A**) Survival curves of HER-2-negative and HER-2-positive (1+/2+/3+) groups. (**B**) Survival curves of HER-2-negative and HER-2 (2+/3+) groups.

**Figure 5:**
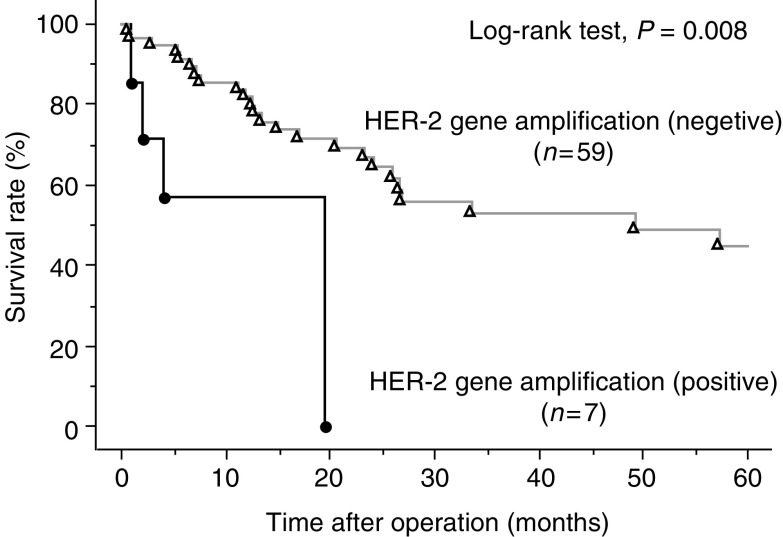
Survival curves of HER-2 gene amplification-negative and HER-2 gene amplification-positive groups.

The survival rate in HER-2-positive (1+/2+/3+) group was significantly worse than that in HER-2-negative group (Figure 4A). Moreover, there was a significant difference in the survival rate between HER-2 (2+/3+) and HER-2-negative group (Figure 4B). The survival rate in the patients with HER-2 gene amplification was significantly worse than that without HER-2 gene amplification (Figure 5).

In univariate analysis, the factors such as HER-2 gene amplification, pT2 and pT3, lymph node metastasis, stages 3–4 were significant prognostic factors for survival (Table 6), although only pT2 factor reached to the significant level as independent risk factors for survival in multivariate analysis (Table 6).

## DISCUSSION

The present study contains several important findings relevant to HER-2 status in oesophageal SCC. First, HER-2-positive tumours (1+/2+/3+) analysed by the HercepTest were observed in 30.3% of all patients and HER-2 gene amplification evaluated by FISH was observed in 11.0% of all patients, of which all IHC (3+) tumours were found to have gene amplification and three out of six tumours with moderate positive (2+) tumours showed gene amplification. Second, HER-2-positive cells existed more diffusely and were larger within each tumour in HercepTest 3+ patients than those who were HercepTest 1+. Thirdly, oesophageal SCC patients with both HLA-A24-and HER-2-positive tumours (1+/2+/3+) accounted for 26% of these cases, and both HLA-A2- and HER-2-positive tumours accounted for 18% of them.

The frequencies of HER-2 overexpression in oesophageal SCC analysed by IHC ranged from 0 to 55.9% (Mori *et al*, 1987; Chang *et al*, 1992; Suo *et al*, 1992, 1995; Shiga *et al*, 1993; Suwanagool *et al*, 1993; Lam *et al*, 1998; Hardwick *et al*, 1997; Akamatsu *et al*, 2003). Furthermore, reports describing HER-2 gene amplification ranged from 0 to 25%, in which these studies were performed by Northern blot, slot blot or RT–PCR analysis (Shiga *et al*, 1993; Ikeda *et al*, 1996; Tanaka *et al*, 1997; Friess *et al*, 1999). This is the first report describing the HER-2 status in oesophageal SCC analysed by two FDA-approved tests, the HercepTest and FISH (PathVysion test). Moreover, there was no previous report describing HER-2 status evaluated by the HercepTest and FISH in relation to the survival rate in oesophageal SCC. As a result, HER-2-positive tumours analysed by the HercepTest were observed in 30.3% of all the patients and HER-2 gene amplification evaluated by FISH was observed in 11.0% of all the patients.

There is increasing evidence that there is a discrepancy in the detection of HER-2 status between the two FDA-approved test, the HercepTest and FISH (PathVysion test), in which the concordance rates ranged from 80 to 90% (Jacobs *et al*, 1999; Varshney *et al*, 2004). There have been several reports that cases with HER-2 overexpression without gene amplification mostly occurred in moderate positive cases (2+) (Perez *et al*, 2002; Varshney *et al*, 2004), in line with this study. Various explanations of this discrepancy have been proposed: transcriptional or post-translational activation (Slamon *et al*, 1989), artifactual high sensitivity of IHC (Varshney *et al*, 2004), the presence of chromosome 17 polysomy (Wang *et al*, 2002) or the low detection rate of FISH analysis (Jacobs *et al*, 1999). We found one case of polysomy in 2+ patients and two cases of polysomy in 1+ patients, suggesting that the presence of chromosome 17 polysomy might be one explanation for the discrepancy between the HercepTest and FISH in oesophageal SCC.

Interestingly, there was one case with HER-2 gene amplification in a HercepTest 1(+) tumour, indicating that the screening of HER-2 status by the HercepTest may underestimate HER-2 gene amplification. Since both the HercepTest and FISH assay have limitations in detecting HER-2 status, both methods should be applied when anti-HER-2 immune targeting, such as Herceptin or cancer vaccination, are considered in oesophageal SCC.

In this study, all the HercepTest 3+ patients had HER-2 gene amplification, and HER-2-positive cells in these cases were present diffusely and were larger within the tumours. Furthermore, the HER-2 expression in HercepTest 3+ patients was also preserved in the metastatic lymph nodes. The action of anti-HER-2-specific CTL correlated to the degree of HER-2 expression on the target tumour cells (Fisk *et al*, 1995; Kono *et al*, 1998). Also, the activity of ADCC induced by Herceptin correlated to the degree of HER-2 expression on the target tumour cells (Kono *et al*, 2002b). These results indicate that HercepTest 3+ patients in oesophageal SCC will be the best candidates for anti-HER-2 immune targeting. It has already been shown in breast cancer trials that there is a greater benefit from Herceptin therapy for 3+ patients compared to those who were 2+ (Cobleigh *et al*, 1999; Slamon *et al*, 2001).

The survival rate in patients with HER-2 expression or HER-2 gene amplification was significantly worse than that without HER-2 expression or HER-2 gene amplification. These results indicated that HER-2 status may be one of the prognostic factors to predict the clinical course of patients with oesophageal SCC, although the HER-2 status did not reach to the significant level as independent risk factors for survival in multivariate analysis in the present study. Since the sample size of the present study is limited, further studies with larger cohorts will be needed to draw valid conclusion.

When considering the cancer vaccination with HER-2-derived peptide epitopes, there is an HLA restriction. It has been shown that HLA-A2- or HLA-A24-restricted peptide epitopes were identified for immunodominant CTL epitopes derived from HER-2 (Fisk *et al*, 1995; Kono *et al*, 1998, 2002b). In the present study, oesophageal SCC patients with both HLA-A24- and HER-2-positive tumours (1+/2+/3+) accounted for 26% of these cases, and both HLA-A2- and HER-2-positive tumours accounted for 18% of them. These populations are considered for cancer vaccination with the HER-2 peptide in oesophageal SCC. In fact, we and others reported that clinical vaccination trials in gastric or breast cancer patients using DCs pulsed with HER-2 peptides confirmed the fact that vaccination with HER-2 peptides is immunogenic, and that HER-2 could be a good target for immunotherapy (Disis *et al*, 1999; Kono *et al*, 2002c). Furthermore, we have recently shown that Herceptin enhances MHC class I-restricted antigen presentation in HER-2-overexpressing tumours, resulting in a higher susceptibility of HER-2-overexpressing tumours to lysis by HER-2-specific CTL (Kono *et al*, 2004). These results suggested that the combination of Herceptin and anti-HER-2-specific CTLs may result in a synergic antitumour effect in oesophageal SCC. Anti-HER-2 immune targeting such as Herceptin or cancer vaccination with HER-2 peptides is novel and attractive approach for oesophageal SCC and the candidates for HER-2-based immunotherapy were limited, but significant populations of oesophageal SCC.

## Figures and Tables

**Table 1:** Clinical features of the patients (*n*=66)

*Age (years old)*	
Mean	65.3
Range	45–81

*Gender*	
Male	62
Female	4

Primary tumour^a^	
pTis	2
pT1a	8
pT1b	18
pT2	5
pT3	32

*LNM*	
Negative	29
Positive	37

*SCC differentiation*	
Well differentiated	15
Moderate differentiated	35
Poorly differentiated	14

*Stage* a	
0	9
I	5
II	25
III	19
Iva	6
Ivb	1

LNM=lymph node metastasis; SCC=squamous cell carcinoma.

a The grade of tumour and stages were defined according to the UICC (TMN) classification.

**Table 2:** Frequencies of HER-2-positive patients detected by IHC in oesophageal SCC

	**Patients (total=66)**
*IHC scores*	
3+	3 (4.5%)
2+	6 (9.1%)
1+	1 1 (16.7%)

IHC=immunohistochemistry; SCC=squamous cell carcinoma.

**Table 3:** Patients with HER-2-positive oesophageal SCC detected by IHC and their FISH analyses

					**IHC**			
**Case number**	**Age (years)**	**Sex**	**Stage^a^**	**Histological^b^ classification**	**Score^c^**	**Pattern**	**Score^c^ of LN**	**FISH**
1	73	M	III	Mod	3+	Diffuse	3+	Cluster
2	54	M	III	Por	3+	Diffuse	3+	Cluster
3	62	M	IVa	Mod	3+	Diffuse	3+	Cluster
4	62	M	IVa	Mod	2+	Spot	0	Cluster
5	59	M	III	Well	2+	Diffuse	0	Cluster
6	57	M	III	Mod	2+	Spot	2+	Polysomy
7	55	M	II	Por	2+	Diffuse	2+	No amplification
8	69	M	II	Well	2+	Spot	No	No amplification
9	76	M	I	Well	2+	Spot	No	Cluster
10	56	M	II	Mod	1+	Spot	0	Polysomy
11	74	M	III	Well	1+	Diffuse	0	Polysomy
12	60	M	III	Mod	1+	Spot	0	No amplification
13	60	M	III	Por	1+	Diffuse	0	No amplification
14	64	M	III	Mod	1+	Diffuse	0	No amplification
15	67	M	III	Mod	1+	Diffuse	0	No amplification
16	71	M	III	Mod	1+	Spot	0	No amplification
17	47	F	II	Por	1+	Diffuse	1+	No amplification
18	74	M	0	Por	1+	Spot	No	Cluster
19	70	M	II	Mod	1+	Diffuse	No	No amplification
20	80	M	II	Por	1+	Spot	No	No amplification

SCC=squamous cell carcinoma; IHC=immunohistochemistry; FISH=fluorescence *in situ* hybridisation; LN=lymph node; No=no lymph node metastasis.

a Stages were defined according to the TNM classification.

b Well=well-differentiated SCC; mod=moderately differentiated SCC; por=poorly differentiated SCC.

c IHC score was defined by the staining intensity of tumour cells (0, 1+, 2+, 3+).

**Table 4:** Correlation between IHC scores and the rate of HER-2-positive tumour cells in each tumour

	**Rate of HER-2/neu-positive cells in each section**		
	**∼30 (%)**	**31–60 (%)**	**61∼ (%)**
*IHC scores*			
3+ (*n*=3)	0	2	1
2+ (*n*=6)	5	0	1
1+ (*n*=11)	8	1	2
	*P*=0.057 by *χ*^2^ test		

IHC=immunohistochemistry.

**Table 5:** Frequencies of the HLA-A haplotype related to HER-2 expression in oesophageal SCC (*n*=50)

	**HER-2 status detected by IHC**	
	**Positive (*n*=18)**	**Negative (*n*=32)**
*HLA-A2*		
Positive (*n*=26)	9 (18%)^a^	17 (34%)
Negative (*n*=24)	9 (18%)	15 (30%) NS^b^

*HLA-A11*		
Positive (*n*=14)	7 (14%)	7 (14%)
Negative (*n*=36)	11 (22%)	25 (50%) NS

*HLA-A24*		
Positive (*n*=32)	13 (26%)	19 (38%)
Negative (*n*=18)	5 (10%)	13 (26%) NS

SCC=squamous cell carcinoma; HLA=human leucocyte antigen.

a Percentage indicates the number of patients out of all patients (*n*=50).

b Not significant by *χ*^2^ test.

**Table 6:** Significance of prognostic factors in univariate and multivariate survival analysis for patients with oesophageal SCC

	**Univariate analysis**			**Multivariate analysis**		
	**Hazard ratio**	**95% CI**	***P*-value**	**Hazard ratio**	**95% CI**	***P*-value**
*FISH*						
Negative	1.0	—	—	1.0	—	—
Positive	4.05	1.31–12.53	0.015	2.94	0.78–11.09	0.111

*HER-2 expression*						
Negative	1.00	—	—	1.0	—	—
Positive	2.06	0.93–4.57	0.074	0.92	0.35–2.41	0.861

*Primary tumour* a						
pTis-pT1b	1.0	—	—	1.0	—	—
pT2	5.85	1.56–21.91	0.009	5.00	1.06–23.52	0.042
pT3	5.28	1.91–14.55	0.001	3.01	0.76–11.95	0.117

*LNM*						
Negative	1.0	—	—	1.0	—	—
Positive	3.13	1.32–7.39	0.009	1.56	0.25–9.68	0.636

*Stage* a						
0–II	1.0	—	—	1.0	—	—
III–IV	5.21	2.33–11.64	<0.0001	2.09	0.30–14.53	0.458

SCC=squamous cell carcinoma; CI=confidence interval; FISH=fluorescence *in situ* hybridisation; LNM=lymph node metastasis.

a The grade of tumour and stages were defined according to the UICC (TMN) classification.
